# Urban retail location: Insights from percolation theory and spatial interaction modeling

**DOI:** 10.1371/journal.pone.0185787

**Published:** 2017-10-04

**Authors:** Duccio Piovani, Carlos Molinero, Alan Wilson

**Affiliations:** 1 Centre for Advanced Spatial Analysis (CASA), University College London (UCL), 90 Tottenham Court Road, London, W1T 4TJ, United Kingdom; 2 The Alan Turing Institute, British Library, 96 Euston Road, London, NW1 2DB, United Kingdom; Beihang University, CHINA

## Abstract

Characterising road networks has been the focus of a large body of research due to it being the main driver of activities in an urban ecosystem and the structuring factor in the dynamics of the city. One of these activities, and one with the largest economical impact in a city, is retail dynamics and its evolution. Therefore, the mathematical modeling of the location of retail activities and of the emergence of clustering in retail centers has as well generated a large number of works. Despite these two interwoven components strongly depending on one another and their fundamental importance in understanding cities, little work has been done in order to compare their local and global properties. Here we compare the road network’s hierarchical structure, unveiled through a percolation analysis of the network, with the retail location distribution defined by exploiting a gravity-based retail model. We interpret the great agreement in the city’s organizations as it emerges from both methodologies as new evidence of the interdependence of these two crucial dimensions of a city’s life.

## Introduction

The study of the spatial organization and the dynamics of retail activities in cities is a long standing problem, one that has puzzled researchers of various fields for a number of years now [1–5]. Despite this multidisciplinary effort fresh approaches have struggled to emerge in the last decades. Recent advances in spatial networks [6, 7], and in particular, in road networks [8–11], combined with the large increase of available data in urban systems, have renewed the interest in the field with efforts aimed at modelling and measuring the formation of retail agglomerations [12–14], and relating it to centrality measurements of the city’s road network [15, 16]. Given its economical importance and the influence that the location of retail activity has on the general structure of a city, and how this structure in turn influences the development of retail centers, we felt compelled to gain insight into the extent to which the road clusters that stem from the percolation process on the road network [17] give rise to retail agglomeration centres [18].

To study and describe retail activities we exploit the retail model introduced in [18], a benchmark in its field. It describes flows of spending power, or money, from population centroids to retail centers. For decades this model has proven itself successful in predicting the dynamics of retail centers [19]. In the model, retail centers compete for the limited amount of resources, represented by the population, and only the more *attractive* and better positioned manage to *survive*. This is elegantly done through an entropy maximizing model [20], which quantifies the aggregate flow from population centroid *i* to retail center *j* with only two parameters: one that sets the scaling between a retailers’ attractiveness and floorspace and another which defines the cost of moving. Indeed, highly visited retail centers grow proportionally to the number of visits, while poorly visited centers shrink in size and are eventually removed from the system. The identity, number and position of retail centers that *survive*, strongly depends on the values of the two parameters, and it has been shown that, by keeping the cost of moving constant and increasing the attractiveness scaling exponent, the model undergoes a phase transition [21–23] from a diverse and heterogenous retail landscape to one where only the most attractive center, by defeating all other competition, manages to survive. In between those two extreme cases the model describes the formation of retail clusters.

This retail model will be put into context with a percolation approach on the road network based on the work presented in [17]. Percolation theory [24] studies the properties of the clusters formed when adjacent sites of a lattice are occupied, and has been applied to study systems in many different contexts, such as oil extraction [25], the study of the electrical conductivity of materials [26], polymerization processes [27], epidemic studies [28], fire spreading [29] and of course urban systems [30, 31]. Moreover, in [17] it has been shown how the road network contains footprints of the socio-economic and cultural evolution of a country and its regions. This has been done by applying percolation theory to the network of the street intersections in the UK, which allowed to clearly uncover regional economical patterns in relation to their infrastructure. In this approach, and very similarly in [32], clusters are the outcome of some thresholding process and reveal a hierarchical organisation of the network. For low thresholds small road clusters scattered through the system appear, which merge with one another by increasing the threshold forming fewer larger clusters, eventually joining in one giant one formed by the whole network.

Understanding the formation of retail centres as one analog to the generation of clusters in the percolation model allows us to establish a comparison between the two approaches. The low threshold scenario corresponds to a small scaling exponent parameter in the model, which forms a heterogeneous and varied retail landscape. While the formation of the giant road cluster obtained by increasing the threshold, corresponds to high values of the model’s scaling exponent which describes the formation of one large retail center. Indeed the scaling parameter and the threshold seem to play the same role in these two clustering processes. It is therefore tempting, to bridge these two formalisms and, following the hierarchical definition that comes from percolation analysis, to interpret the formation of retail clusters described by the model as a fingerprint of a hierarchical organization in the economic activities of a city. As we will show in great detail the two approaches describe a very similar urban hierarchical structure with the location and size of the retail and road clusters being in great agreement. This result seems to bring new evidence on the polycentric organisation of the city, and indeed sheds new light on the relationship between the road network of a city and the economical activities that develop on it.

## Materials and methods

In this section we will go through the details of the procedures we want to compare, and the data used both for calibration and for testing. We will start by defining the retail model, and analysing its main results, we will then present the application of the percolation process to London’s road network, and finally present the data.

### The retail model

By following the procedure outlined in [23], we can define the flows from population centroids *i* to retail centres *j* as described by the equation

$$\begin{array}{c} T_{ij}=\frac{p_{i}w_{j}^{\alpha }\text{exp}\left(-\beta c_{ij}\right)}{Z_{i}} \end{array}$$ (1)

where *p*_*i*_ is the population in the origin *i*, *w*_*j*_ is the aggregated floorspace of retail center *j* and *c*_*ij*_ the cost of moving from *i* to *j*, that we will simply quantify as the euclidean distance between the two points. We can see how these flows are defined by two parameters, namely *α*, which sets the scaling between the *attractiveness* and the floorspace of a retailer and *β* which tunes the *cost of moving*. *Z*_*i*_ is the normalisation factor, which under the constraint of the total outflow being equal to the population, i.e. ∑_*j*_ *T*_*ij*_ = *p*_*i*_, becomes

$$\begin{array}{c} Z_{i}=\sum_{k}w_{k}^{\alpha }\text{exp}\left(-\beta c_{ik}\right) \end{array}$$ (2)

As one can see in [23], the form of the flows in Eq (1) comes out of an entropy maximising process, and is obtained under the constraints that come from the observed data: the population’s *p*_*i*_, the aggregated floorspace’s *w*_*j*_ and the cost matrix’s *c*_*ij*_ spatial distribution. This means that the set of flows {*T*_*ij*_} are an equilibrium configuration, that depends on the input data as well as on the values of the parameters *α* and *β*. Any small change in the input data would yield a rapid reconfiguration to a new attractor state. We can therefore interpret this process as a *fast dynamics* one.

Moreover we can, by exploiting Eqs (1) and (2), predict the evolution of the floorspace distribution {*w*_*j*_}, considered constant during the *fast dynamics*. By calculating the total inflow to retail centre *j* as *d*_*j*_ = ∑_*i*_ *T*_*ij*_, we define the dynamics equation as

$$\begin{array}{c} \Delta w_{j}=\epsilon \left(\kappa d_{j}-w_{j}\right) \end{array}$$ (3)

**Fig 1:**
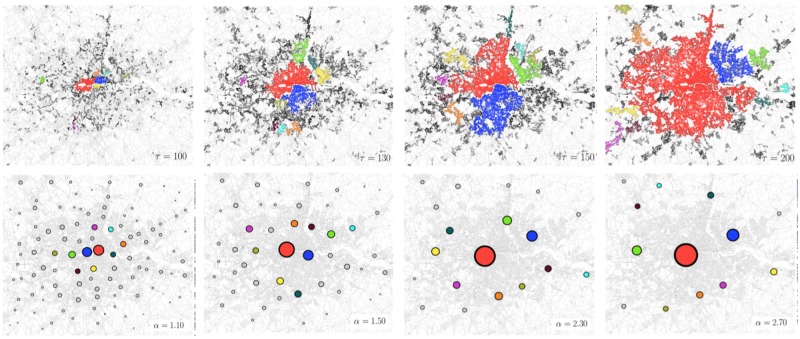
The figures in the top panel represent the evolution of the road clusters for growing values of the threshold *τ*. The figures in the bottom panels show the distribution of the equilibrium configuration defined in Eq (3), *w*^eq^(*α*, *β*), for growing *α*’s and fixed *β* = 0.8. The size of the circles are proportional to the total floorspace of the retail cluster. In both cases the colours indicate the rank of the clusters. There is a striking similarity both in the types of dynamics, and in the spatial distribution.

The set of equations in Eq (5) are complicated non linear equations that can only be solved iteratively., because every variation in any retailer’s floorspace *w*_*k*_, modifies all other equations. The *ϵ* is a small factor that allows the iterative process to converge. Therefore we have

$$\begin{array}{c} w_{j}\left(n+1\right)\leftarrow w_{j}\left(n\right)+\Delta w_{j}\left(n\right) \end{array}$$ (4)

with *n* being the current iteration. Eqs (3 and 4) tell us that *w*_*j*_ will increase if *κd*_*j*_ > *w*_*j*_ and shrink in the opposite case. The constant *κ* is there to make sure that all quantities are measured in commensurate units, and converts the flow of people into floorspace. Its value must be calibrated on the data. The solution to Eq (3), is given by the set of equations $\kappa d_{j}=w_{j}^{\text{eq}}$ which explicitly become

$$\begin{array}{c} \kappa \sum_{i}\{\frac{p_{i}{\left(w_{j}^{\text{eq}}\right)}^{\alpha }\exp\left(-\beta c_{ij}\right)}{\sum_{k}{\left(w_{k}^{\text{eq}}\right)}^{\alpha }\text{exp}\left(-\beta c_{ik}\right)}\}=w_{j}^{\text{eq}} \end{array}$$ (5)

which implies that $\Delta w_{j}^{\text{eq}}≃0\;\;\forall j$. The model we have just defined has a rich behaviour and describes different types of retail structures {*w*^eq^}, according to the two parameters *α* and *β*. For *α* > 1 larger shops will be more attractive and a small *β* implies higher probabilities of interaction over longer distances to achieve the benefits of size. Hence large *α* and small *β* combinations generate structures with a small number of large *w*_*j*_ centres and vice versa. In the bottom panels of (Fig 1) we can see how, by fixing *β* = 0.8 and increasing *α*, the number of retail centres decrease with the ones remaining becoming larger and larger.

### Percolation on London’s road network

In this work we apply a percolation process to London’s road networks in order to uncover its hierarchical structure, following the procedure used in [17]. In the approach, the nodes of the weighted network are the road intersections, while the links are each road joining two intersections. These are weighted by their lengths, so two crossings connected by a long road will have a link with a high weight connecting them and vice versa. The approach undertaken to calculate the percolation of London’s road network consists of the following steps: we begin by setting a threshold *τ*, then we select every link who’s weight falls below that threshold, *r*_*ij*_ < *τ* and extract the subgraph formed by those links. The weakly connected components of the subgraph are the clusters of the network generated by the percolation process for a given threshold. The clusters are constructed such that they have at least a link connecting them with a weight smaller than the given threshold. These clusters form a tree structure given that for two thresholds *τ*_1_ and *τ*_2_, if *τ*_1_ < *τ*_2_, a cluster generated using *τ*_1_ will be completely contained into a cluster obtained using *τ*_2_. This allows us to construct a hierarchical tree that follows the ordering of the regions induced by the percolation which uncovers the intrinsic structure of the system.

**Fig 2:**
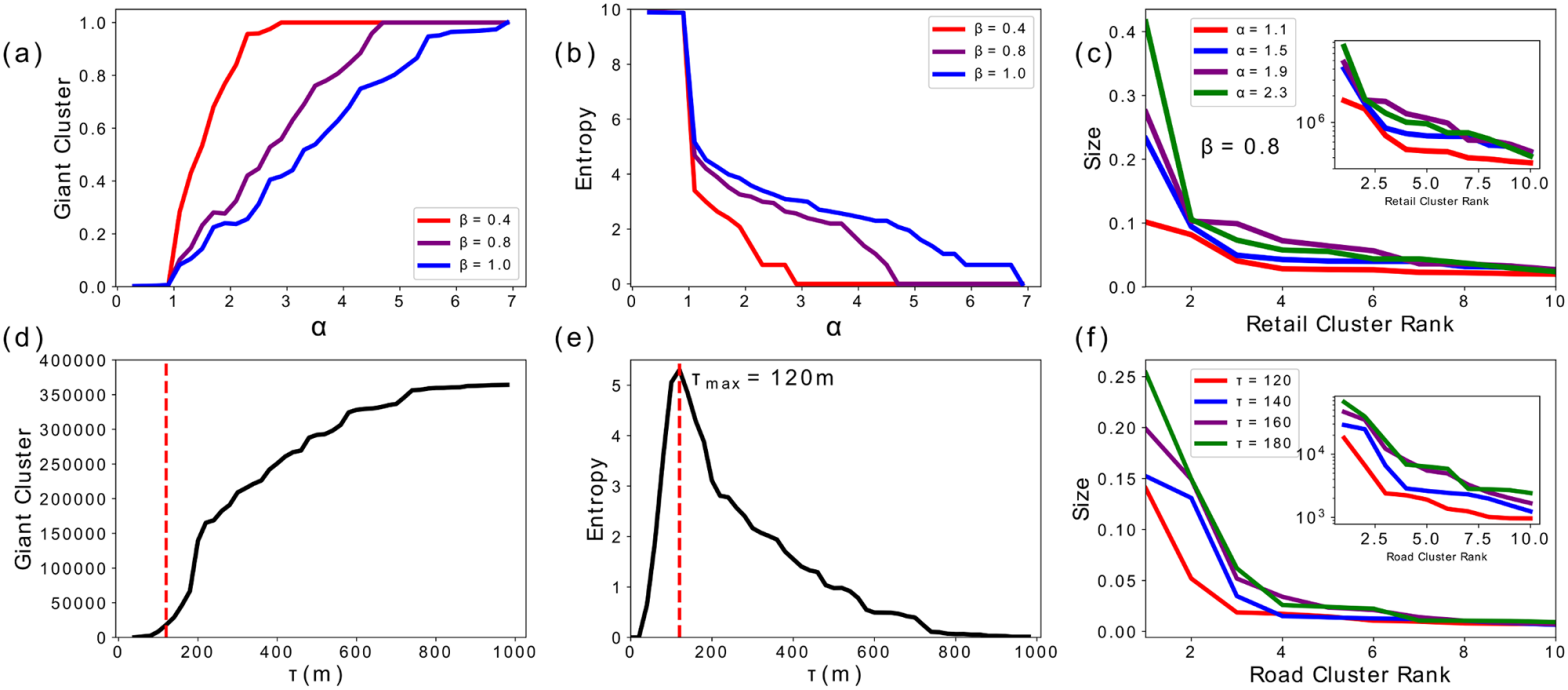
The top panels refer to the retail dynamics with *β* = {0.4, 0.8, 1.0}, while the bottom panels to the hierarchical percolation. (**a**)-(**d**) The evolution of the size of the giant cluster, is presented, for increasing values of *τ* and *α*. (**b**)-(**e**) We show the evolution of the spatial entropy. Both quantities show a very similar behaviour in the two approaches. (**c**)-(**f**) We can see the distribution of the cluster (*β* = 0.8 for the retail model) that have an exponential form as shown in the insets.

Percolation is a critical process that, generally, presents a phase transition at a critical probability (in our case threshold). The term phase transition here is used perhaps freely used since in the type of percolation that we are dealing with, this phase transition is not as clear as in other percolation processes. As we can see in (Fig 2e) the threshold that generates the maximum entropy configuration is *τ* = 120 and that is the point where the clusters are simultaneously maximizing their sizes while equilibrating their difference. Below it, the clusters are small, while above it the giant cluster starts to take over the whole distribution. It is therefore, the threshold with a larger information in terms of the distribution of the cluster sizes.

We must clarify that the specific value of the threshold *τ*_max_, for which the maximum entropy configuration is generated, depends on the value of the minimum cluster size accepted in the analysis *n*_min_. For the purpose of this work we have arbitrarily set this value to *n*_min_ = 100, but the results we have obtained can be extended to any other value of this parameter, with no loss of generality.

Given a threshold *τ* the clusters of the percolation separate the network into regions that have a similar density of intersections, and it has been shown in [33] and in the supplementary information of [34] that this is correlated with the population density. This is probably due to the fact that buildings have to be located next to a road intersection. Moreover it is safe to assume that retailers tend to position themselves close to population centers proportionally to their size. We can therefore interpret the study undertaken in this paper as an effort to quantify this effect through the comparison between retail and intersection clusters.

## Results

As explained in the introduction, our aim is to compare the location and size of road clusters, that come from a percolation analysis of the road network, and the retail clusters described by the model as solutions of Eq (3). Moreover we want to compare the role played by the scaling parameter *α* of the model in the formation of retail clusters with that played by the threshold *τ* in the percolation.

To do so we will start with a qualitative observation of the two evolutions: in (Fig 1) we show the evolution of the spatial distribution of the clusters in the two approaches. The figures in the top panel show the evolution of the percolation clusters, where nodes of the same colour belong to the same cluster, and where the colour indicates the rank of the cluster. White nodes are those that either don’t belong to any cluster or they belong to a cluster who’s size is smaller than 100 (minimum cluster size). We consider white nodes as not belonging to the system for the given threshold, making the fraction of *active* nodes dependent on *τ*. In the bottom panels, following the same logic, we present the {*w*^eq^} configuration that comes out of Eq (3), where we have fixed the *β* parameter to 0.8 and where we vary the value of *α*. We see how for every new value of *α* the configuration {*w*^eq^} is formed by retailers that vary in number, location and size. At a first glance we can see how by increasing *τ* in the road percolation approach and increasing *α* in the retail model the behaviour is very similar: in both cases the number of cluster decreases while their size tends to increase, and high ranked clusters tend to position themselves in similar positions.

For a more quantitative comparison of the macroscopic properties of the two evolutions we study the size of the *giant* cluster and of the entropy of the cluster sizes for increasing values of *α* and *τ*. To calculate the entropy we have used Shannon’s formula

$$\begin{array}{c} H_{\tau ,\alpha }=-\sum_{x}p_{\tau ,\alpha }\left(x\right)\log p_{\tau ,\alpha }\left(x\right) \end{array}$$ (6)

where *x* runs on the cluster sizes and *p*(*x*) is the probability of finding a cluster of size *x*, for each value of *τ* and *α*. In (Fig 2) we can see how the evolution of these two quantities follows the same behaviour in both approaches. In the percolation on the road network (bottom panel) (Fig 2d) and (Fig 2e), for low values of *τ*, increases in the threshold imply increases in the entropy. This corresponds to a slow increase in the size of the giant cluster. Around *τ* = 130 the Entropy reaches its maximum and we can see a change in the curvature of the giant cluster which starts a steeper increase. From then on, as one may expect, the entropy of the system decays to zero and the giant cluster *spreads* to the whole network.

In (Fig 2a and 2b) we show how the clusters that form during the dynamics of the retail model follow a very similar dynamics. In this case, for all values of *β*, both the decrease in the entropy as well as the increase in the *giant cluster* size are very slow for *α* < 1. This is due to the fact that for *α* < 1 the model does not describe the formation of retail clusters, and the equilibrium configuration {*w*_eq_} is very similar to the initial condition found in the data. That said, one can see a clear transition happening at *α* = 1, where the entropy rapidly starts decreasing. This behavior does not depend on the value of *β*, although higher values of the parameter yield smoother transitions while for lower values we get sharper ones. However, no matter the value of *β* the system always ends up with the same winner: the same retail cluster manages to outplay the rest of the competitors and have all the flows in the systems directed towards it. This means that for each value of the parameter we are always observing the same transition, which begins from the same initial condition and ends up in the same *ground state*. We could think of *β* as setting the scale of the transition, in terms of *α*: the system always *explores* the same states, but in a low *β* scenario one needs more coarse grained values of *α* than in a high *β* system to actually observe them all. This allows us to fix the value of *β* (we will arbitrarily set to *β* = 0.8) and study the behaviour of the system only varying the values of *α*. Given our interest in understanding the spatial distribution of retail clusters with respect to road clusters, we will concentrate our analysis on *α* > 1 values. In the two figures on the right (Fig 2c and 2f) we show the distribution of the sizes of the clusters, i.e. the aggregated floorspace for the retail centres and number of nodes on the road network clusters, of the 10 largest clusters. In the insets we can see how the distribution has an exponential form in both cases, and how increasing *α* and *τ* has the same effect on the distribution, namely increasing its steepness.

In this paragraph we have seen how at a macroscopic level the two approaches describe a very similar dynamics in the formation of clusters, and eventually of a *giant cluster*. We have also shown how we can fix *β* without loss of generality in the results and how, in the retail model, the *α* parameter plays the same role as the threshold *τ* plays in the percolation. In the following we will measure the spatial similarity of the cluster’s distribution.

### Retail distribution on road clusters emerging from the percolation process

Before moving to a more detailed comparison of the spatial distribution of the two types of clusters, we must take a step back, and see how the retailers found in the data are distributed on the percolation road clusters. This step is interesting for two reasons: on one hand it will tell us if we can learn something on the real retail distribution by analysing its relationship to the road clusters, and on the other hand it will serve as a benchmark to then better quantify the effects of the retail model’s dynamics. In [17] the authors have shown how starting from the road network of the whole of the UK, the cities emerged as clusters of the road network for *τ* = 300*m*. Given that our analysis is applied at the city level in London, we will take that as our maximum threshold.

As already mentioned in the previous section, in the VOA dataset, where we find the information on the retailers, retailers are aggregated at the post-code level, which in the UK and therefore in the city of London, differs for every street. Therefore retailers that share the same street have identical x, y coordinates in the dataset. We therefore begin our analysis by aggregating all retailers with the same coordinates, and assigning their floorspace to the nearest node of the road network. In other words this consists in allocating the street’s retail floorspace to the road intersection closest to the street’s centroid. For each value of the threshold *τ* we then study the fraction of floorspace assigned to the emerging clusters. To do so we compare the fraction of nodes of the road network that form the clusters for given threshold *τ*, namely

$$\begin{array}{c} n_{\text{in}}\left(\tau \right)=\frac{n_{\text{c}}\left(\tau \right)}{N_{\text{road}}} \end{array}$$ (7)

where *n*_c_(*τ*) is the sum of nodes that belong to a percolation cluster of the system and *N*_road_ the total number of nodes in the system, to the amount of retail floorspace *contained* in them:

$$\begin{array}{c} f_{\text{in}}\left(\tau \right)=\frac{f_{\text{c}}\left(\tau \right)}{F_{\text{tot}}} \end{array}$$ (8)

where *f*_*c*_(*τ*) is the amount of floorspace assigned to the nodes of percolation clusters and *F*_tot_ the total amount of floorspace in the system. We can then study their difference

$$\begin{array}{c} \Theta \left(\tau \right)=\left(n_{\text{in}}-f_{\text{in}}\right) \end{array}$$ (9)

A Θ ≃ 0 case would indicate a random spatial distribution of the retailers on the road network. Meaning that the distribution of retail floorspace on the road network would be independent of the hierarchy indicated by *τ*, and one would obtain the same *f*_in_ by selecting the same fraction of nodes, *f*_in_, using any other criteria. On the other hand Θ < 0, would indicate a tendency of retailers to be on roads that are not yet in the system, while the opposite case Θ > 0 would unveil a spontaneous tendency of retailers in positioning themselves on highly connected clusters.

Once again, in order for this analysis to make sense, we have to set a minimum size for the road clusters. Without it, and considering a single node as a cluster of size 1, all the retail floorspace would be considered completely contained for any value of the threshold *τ*. In that case *f*_in_ = *n*_in_ = 1 ∀*τ*. As mentioned in the previous section we will show the results obtained with *n*_min_ = 100, but have tested other thresholds, and despite slight numerical differences, we have found the same behaviour.

**Fig 3:**
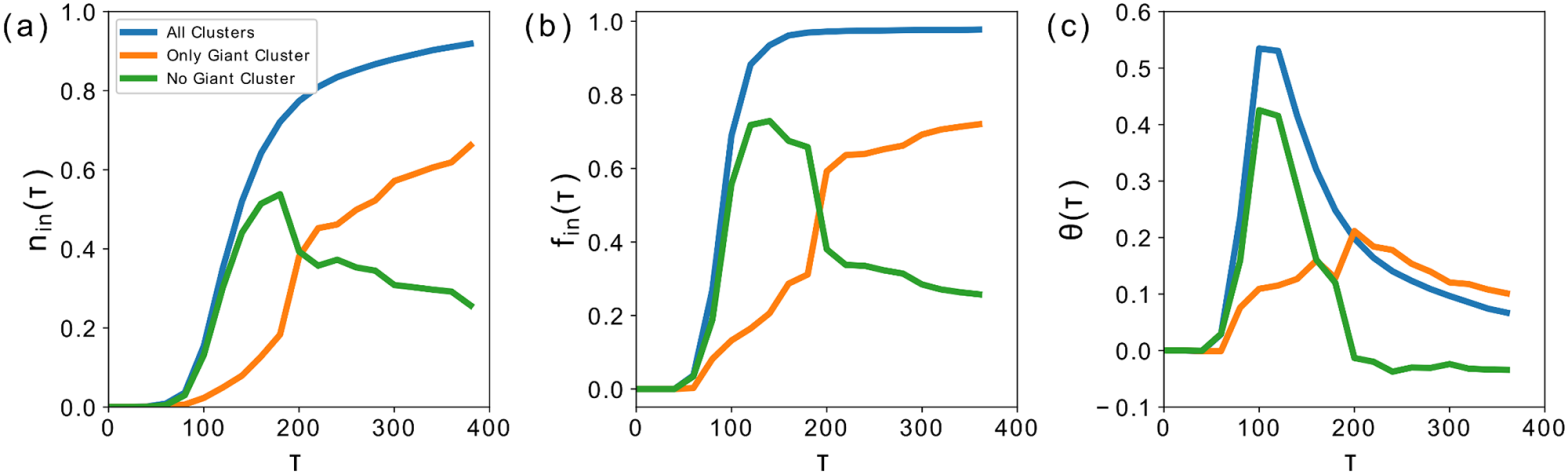
(**a**) This figure represents the evolution of the fraction of nodes of the road network allowed in the system for the given value of *τ*, *n*_in_(*τ*) (black curve). Moreover we show the same quantity considering only the giant cluster (blue curve) and every cluster but the giant cluster (yellow curve). (**b**) Here we show the fraction of the total retail floorspace belonging to the nodes that form the clusters *f*_in_(*τ*) where the colors have the same meaning. (**c**) We compare the two curves by showing their difference Θ(*τ*) = (*n*_in_ − *f*_in_).

In (Fig 3) we show the behaviour of the distribution of the three quantities *n*_in_(*τ*), *f*_in_(*τ*) and Θ(*τ*) in (Fig 3a, 3b and 3c) respectively. We have measured the quantities on the full network (black curve), the network without the giant cluster (yellow curve) and only considering the giant cluster (blue curve). This has been done to make sure the results were not being dominated by the giant cluster. By comparing the figures we can see how *f*_in_ grows much faster in *τ* with respect to *n*_in_, and we constantly get Θ > 0. Furthermore the yellow curve shows that up to *τ* = 200 this is true even if we exclude the giant cluster from the analysis. If it is clear from these results that retailers tend to position themselves in central locations, what also emerges is a tendency to choose highly connected clusters, or in other words clusters formed by a dense grid of alleys and road crossings. An in depth analysis of these results would require a study of its own and we leave it to future research. We will now exploit these results to understand the effects introduced by the dynamics described in Eqs (1)–(5).

### Comparing the spatial distribution of retail and road clusters

We have seen how the retailers are more likely to be found on roads that belong to very connected clusters, but we still did not apply the model’s dynamics to the retailer’s spatial distribution. We have also seen that just like in the road network percolation *τ* shows the hierarchical relationship of the roads, *α* in the model describes the formation of retail clusters described in {*w*^eq^}. Now we want to study the analogies and differences of these emerging structures. We do this by repeating the same analysis we have done on the full data set, this time on the equilibrium configurations that comes out of Eq (3). Of course the introduction of the *α* adds a new degree of freedom, and now *f*_in_ ≡ *f*_in_(*α*, *τ*) and Θ ≡ Θ(*α*, *τ*).

**Fig 4:**
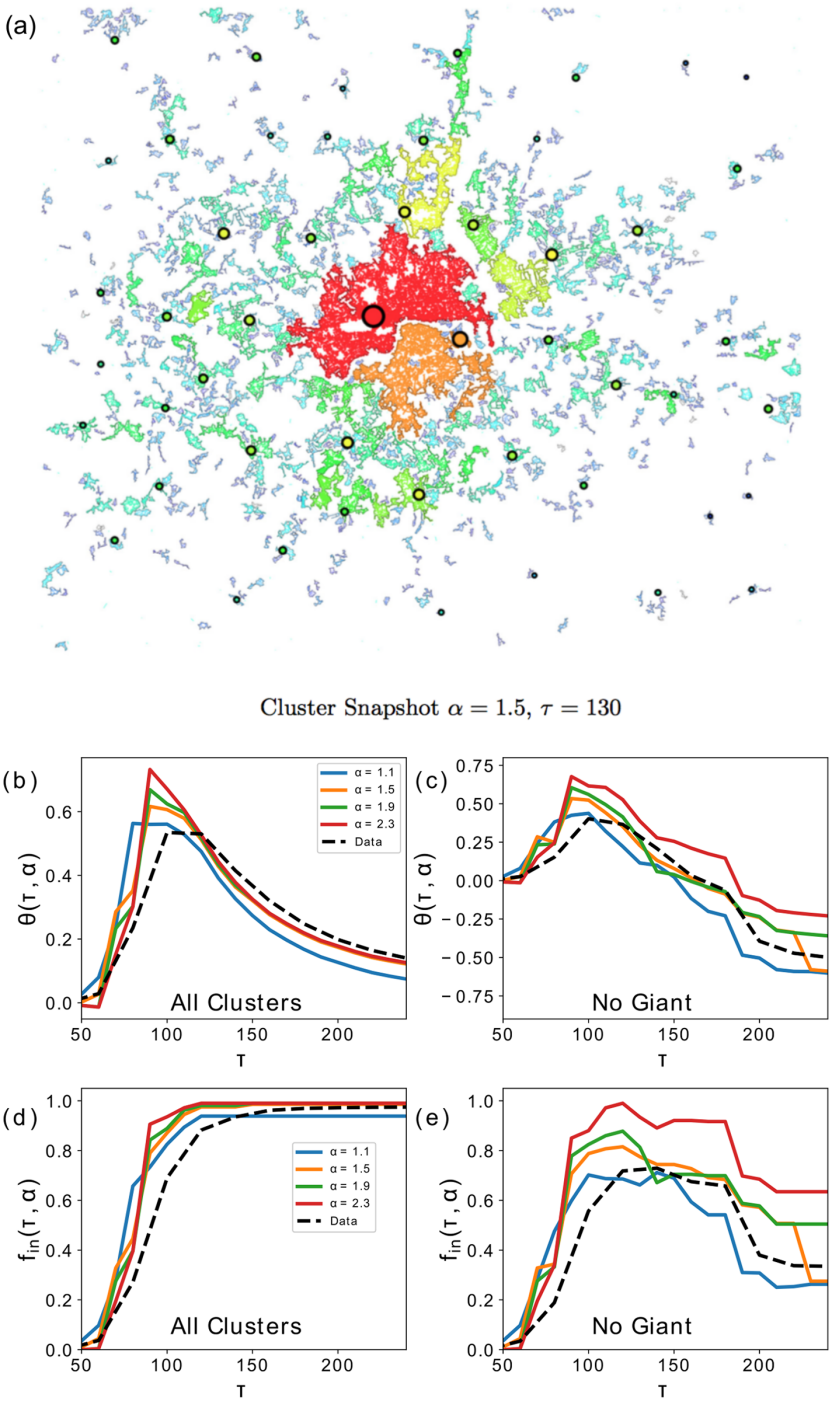
(**a**) We have overlapped a snapshot of the road network clusters for *τ* = 130 with the retail clusters {*w*^eq^} for *α* = 1.5. The colours indicate the size of the clusters in a logarithmic scale. We can see how most of retail clusters fall on road clusters, and there is good agreement between the spatial distribution of the ranks. In (**b**)-(**c**) We show how Θ(*α*, *τ*) varies with *τ* for several values of *α*, on the full network and not considering the giant cluster. The dashed black line indicates the values obtained from the data. We can see that the model for *α* ≤ 1.9 constantly produces higher values, with and without the giant cluster, while for *α* = 2.3 we can see how the giant cluster plays a fundamental role. This is because for that value of *α* the floorspace is mostly concentrated in the giant cluster (see Fig 2). (**d**)-(**e**) We show the *f*_in_(*α*, *τ*) with and without the giant cluster. The results are in line with that said for the previous figures.

In (Fig 4a) we start by showing an overlap of the clusters obtained with *β* = 0.8, *α* = 1.5 and *τ* = 130. At a first glance we can see how big retail clusters tend to lay on big road clusters, and vice versa, and how this is true even for clusters appearing at the periphery of the city. Some clusters that did not exactly overlap lie one next to the other. To quantify this impression, in (Fig 4b and 4d) we show *f*_in_(*α*, *τ*) and Θ(*α*, *τ*) for values of *α* ranging from 1.1 to 2.3. For *α* greater than that, the floorspace is mainly contained in the giant cluster which dominates any analysis. Perhaps surprisingly *f*_in_(*α*, *τ*)>*f*_*in*_(*τ*) and Θ(*α*, *τ*)>Θ(*τ*) ∀*τ*, indicating that retailers belonging to the clusters have *survived* the dynamics more than those not belonging to the clusters, and have grown in size. This is true both if we include the giant cluster and if we leave it out. For *α* = 2.3 however 40% of the retail floorspace is concentrated in the retail giant cluster which lies on the road giant cluster, therefore not considering it ruins the results.

**Fig 5:**
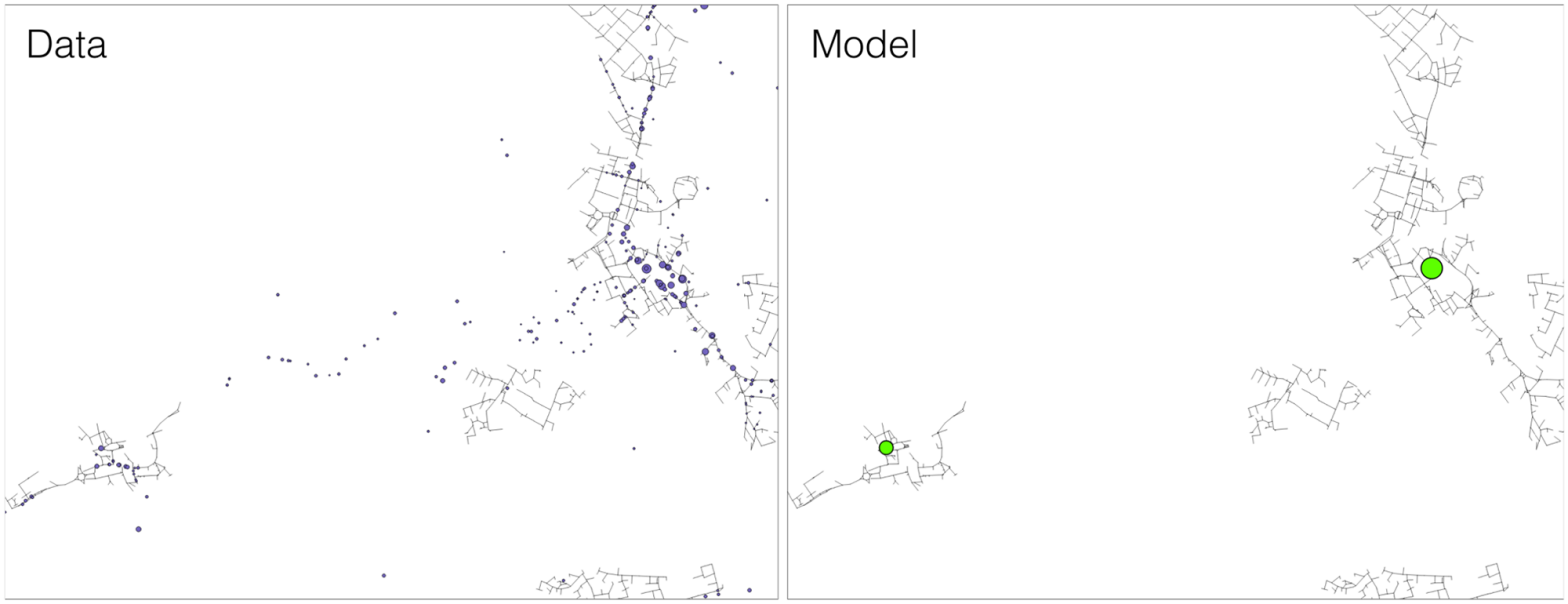
A diagram of the effect of the model’s dynamics on the retail floorspace distribution. We see how the retail floorspace distribution in the data falls outside the road cluster, and how after the dynamics, the floorspace is all concentrated inside the cluster.

The point we are trying to make is that the retailers that survive the model’s dynamics {*w*_eq_}, tend to belong to road clusters. This effect is not given by the actual configuration of retail floorspace in the system, and we can see in (Fig 4), by comparing the results with the black dashed line, that this tendency is increased by the model. The effects of the model’s dynamics on the floorspace distribution are shown in the diagram in (Fig 5a). By increasing the *α* parameter the model describes a retail activity concentrated in fewer larger retail clusters, which we have showed have a tendency to be positioned on nodes that belong to road clusters, for every *τ*. The high level of agreement between the two formalisms with no apparent relationship hints to the existence of an underlying common mechanism in the two methodologies. The high level of agreement between the two formalisms with no apparent relationship hints to the existence of an underlying common structure. Our understanding is that this stems from the locations of the population centres which, as we have seen in [33] and in the supplementary information of [34], have a very strong correlation with the location of road intersections which in turn attract retail activity. Indeed in the case of the retail model, the population is introduced as an input to the dynamics and it is related to the size of the emerging clusters through the cost term *c*_*ij*_, and retailers that survive the model’s dynamics in average are closer to the population centres. Therefore the clusters formed by the density of intersections at the road level are implicitly picking up this densely populated areas as do the model’s retail clusters.

## Conclusion

We can find throughout the literature approaches that characterise cities through the analysis of their road network [8, 9, 11, 35] and others that study the distribution and nature of retail activity [18, 36, 37]. Furthermore, some work has been done in order to relate these two approaches [15, 16] and the contribution presented in this paper goes in that direction. Moreover, the presence of a clear hierarchical structure in road networks has been shown [17] as has the theoretical mechanism that leads to the formation of this hierarchy [7]. Percolation theory has proven itself as a useful tool to study urban areas [17, 38] but to our knowledge no work has been done to export these concepts to analyse the organisation of retail activities in cities. In order to do so we have used the single constrained gravity model [18], perhaps the most widely used model of the field, and characterised its results by *embedding* them on the city’s road network through the clusters generated by the percolation process. We have used the city of London as a test case, and given the quality of our results now plan to extend our research to the whole of the UK. In other words we have presented an attempt to relate the different configurations of clusters obtained through purely geometrical means based on the road network, with the location of retail clusters described by the retail model. We have seen how the location and size of the clusters depend on the threshold *τ*, in the road network, and the scaling exponent *α* in the model: by conveniently tuning these two parameters we are able to reproduce an almost identical spatial configuration. The city’s hierarchical organization that emerges from these two approaches is in complete agreement. We believe that the results presented in this paper are important for a number or reasons. Namely, we have bridged the results of a model first presented many years ago with an approach that only recently has been applied to study urban spaces, and comparing the results we have interpreted them in a new way. In doing so, we have brought evidence to the existence of a hierarchical spatial organization of retail activity in London, and believe this result can be very useful in future modeling.
